# microRNA-302c-3p inhibits renal cell carcinoma cell proliferation by targeting Grb2-associated binding 2 (Gab2)

**DOI:** 10.18632/oncotarget.15463

**Published:** 2017-02-17

**Authors:** Dong-Hua Gu, Jia-Hui Mao, Xiao-Dong Pan, Hua Zhu, Xinfeng Chen, Bing Zheng, Yuxi Shan

**Affiliations:** ^1^ The Department of Urology, The Second Affiliated Hospital of Soochow University, Suzhou, China; ^2^ The Department of Urology, The Second Affiliated Hospital of Nantong University, Nantong, China; ^3^ Department of Pathophysiology, Nantong University School of Medicine, Nantong, China

**Keywords:** renal cell carcinoma (RCC), Grb2-associated binding 2 (Gab2), microRNA-302c-3p, Akt and cell proliferation

## Abstract

The expression and biological function of Grb2-associated binding 2 (Gab2) in renal cell carcinoma (RCC) cells was tested here. We showed that Gab2 expression was significantly elevated in human RCC tissues and RCC cells. It was correlated with over-activation of Akt and downregulation of microRNA-302c-3p (“miR-302c-3p”), a putative Gab2-targeting microRNA. Knockdown of Gab2 inhibited Akt activation and 786-O RCC cell proliferation. Reversely, forced over-expression of Gab2 led to Akt hyper-activation to facilitate 786-O cell proliferation. Exogenous expression of miR-302c caused Gab2 downregulation, Akt inhibition and 786-O cell proliferation inhibition. On the other hand, miR-302c-3p depletion by expressing its anti-sense (“antagomiR-302c”) led to Gab2 upregulation, Akt activation and increased 786-O cell proliferation. Significantly, miR-302c-3p failed to affect the proliferation of 786-O cells with shRNA-depleted Gab2. Together, we suggest that miR-302c-3p depletion in human RCC cells leads to Gab2 over-expression, Akt hyper-activation and cell proliferation.

## INTRODUCTION

As a common renal cancer, renal cell carcinoma (RCC) causes large mortalities each year [1–4]. RCC's incidence has been rising in China and around the world [1–4]. Many RCCs are diagnosed at late stages, often with local/systematic metastasis. Therefore the prognosis of RCC has been poor [1–3, 5, 6]. The curable surgery resection of RCC is only suitable for few patients with early-stage tumors [1, 7–9]. Our group [10–12] has been dedicated to exploring novel oncotargets for RCC.

The mammalian Grb2-associated binding (Gab) scaffolding/adapter family proteins have three highly conserved members, including Gab1, Gab2, and Gab3 [13–16]. Gab family proteins are composed of a N-terminal PH domain, a tyrosine residue domain and a proline-rich domain [13–16]. They are known to interact with signaling proteins with SH2 and SH3 domains, and to consequently form multi-molecular signaling complexes [13–16]. For example, Gab proteins could dock with PI3K p85 to mediate Akt activation [17, 18]. Hyper-activation of Akt signaling is a hallmark of RCC, which is extremely important for cancer cell survival, proliferation and chemo-resistance [19, 20]. Meanwhile, Gab could also interact with SHP2 to mediate Erk-MAPK activation [18, 21]. To our best knowledge, the expression and potential biological functions of Gab2 in RCC have not been examined thus far. Here, we show that Gab2 is over-expressed in human RCC tissues and RCC cells, which is important for Akt activation and RCC cell proliferation.

Accumulative studies have proposed that microRNAs are important in progression of RCC and other malignancies [22–26]. Dysregulation of microRNAs is now recognized a characteristic of cancer [26]. Through searching multiple microRNA databases, a putative Gab2-targeting microRNA, microRNA-302c-3p (“miR-302c-3p”), was identified. We here provided evidences to suggest that miR-302c-3p downregulation in human RCC cells causes Gab2 over-expression, Akt hyper-activation and cell proliferation.

## RESULTS

### Gab2 over-expression correlates with miR-302c-3p downregulation and Akt hyper-activation in RCC tissues and RCC cells

**Figure 1:**
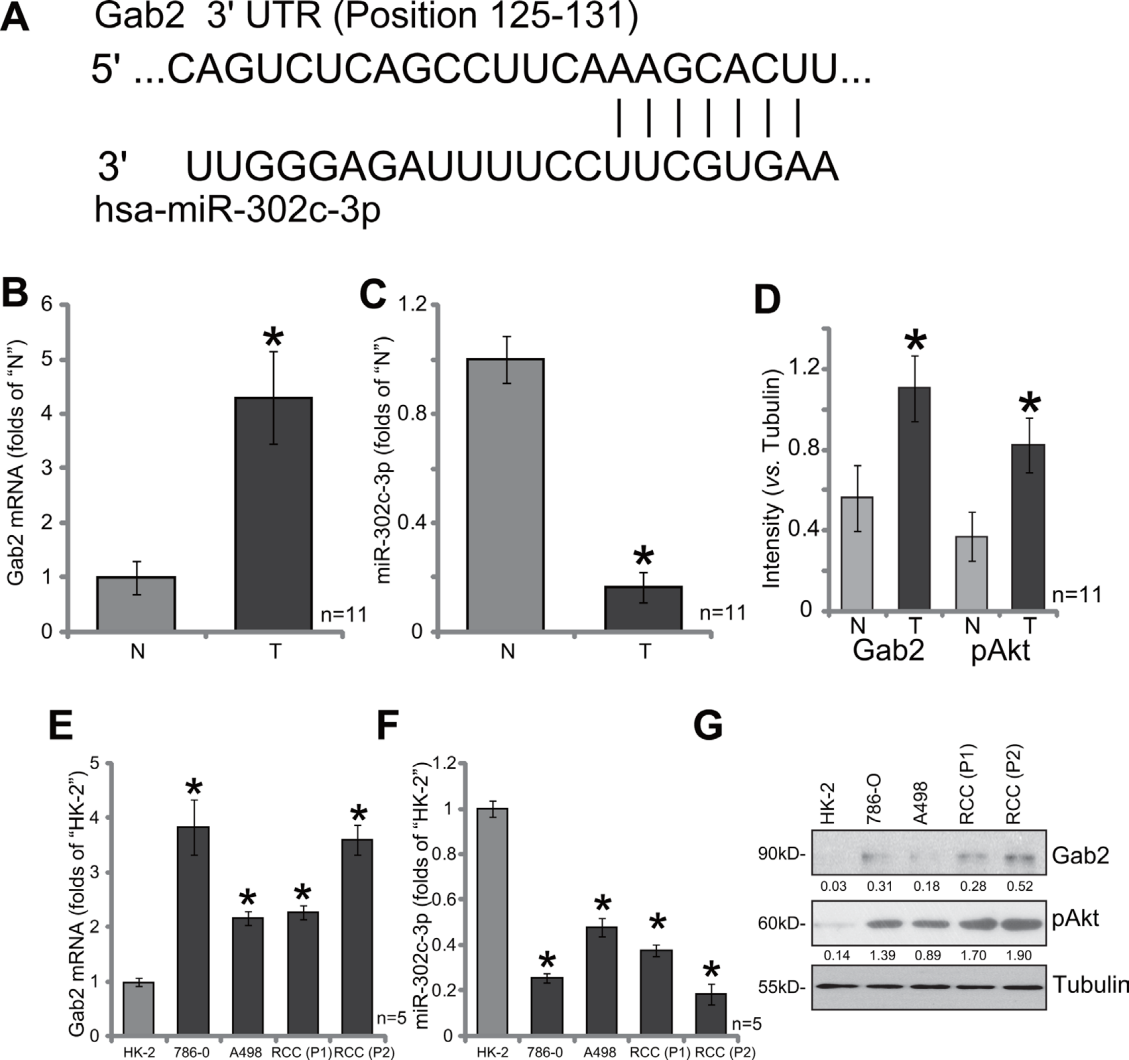
Gab2 over-expression correlates with miR-302c-3p downregulation and Akt hyper-activation in RCC tissues and RCC cells (**A**) miR-302c-3p putatively targets the 3′ untranslated regions (UTR) of Gab2 (at position 125-131). A total of eleven (11) human RCC tumor tissues (“T”) and their paired surrounding normal renal tissues (“N”) were subjected to quantitative real-time PCR (“qRT-PCR”) assay to test expression of Gab2 mRNA (**B**) and miR-302c-3p (**C**); Listen protein expression was tested by Western blot assay, data were quantified (**D**). Expressions of Gab2 mRNA (**E**), miR-302c-3p (**F**) and listed proteins (**G**) in established human RCC cell lines (786-O and A498), primary human RCC cells [RCC (P1) and RCC (P2)] and HK-2 tubular epithelial cells were tested. Gab2 and p-Akt expressions were quantified (*vs*. Tubulin, G). *p < 0.05 *vs*. “N” group (B, C and D) or HK-2 cells (E and F).

As illustrated in Figure 1A, the miR-302c-3p putatively targets the 3′ untranslated regions (UTR) of Gab2 (at position 125–131). We thus tested expression of Gab2 and miR-302c-3p in the human RCC tissues. A total of eleven (11) human RCC tumor tissues (“T”) along with their paired surrounding normal renal tissues (“N”) were analyzed. Quantitative real-time PCR assay (“qRT-PCR” assay) results showed that Gab2 mRNA level was significantly elevated in the RCC tissues, as compared to that in the normal renal tissues (Figure 1B). Reversely, miR-302c-3p level was downregulated in RCC tissues (Figure 1C). Western blot assay was employed to test Gab2 protein expression. Quantified results in Figure 1D confirmed Gab2 protein upregulation in RCC tissues, which was correlated with Akt hyper-activation (p-Akt at Thr-308).

We also tested expression of Gab2 and miR-302c-3p in human RCC cells, including two established RCC cell lines (786-O and A498) [10–12] as well as two primary human RCC cell lines: “RCC (P1)” and “RCC (P2)”. As compared to the HK-2 tubular epithelial cells (non-cancerous renal cells), Gab2 mRNA upregulation (Figure 1E) and miR-302c-3p depletion (Figure 1F) were observed in the above RCC cells. Meanwhile, Gab2 protein upregulation and Akt over-activation were also observed in RCC cells (Figure 1G). Collectively, Gab2 expression is elevated in human RCC tissues and cells, which is correlated with miR-302c-3p downregulation and Akt over-activation.

### Gab2 shRNA knockdown inhibits Akt activation and 786-O RCC cell proliferation

**Figure 2:**
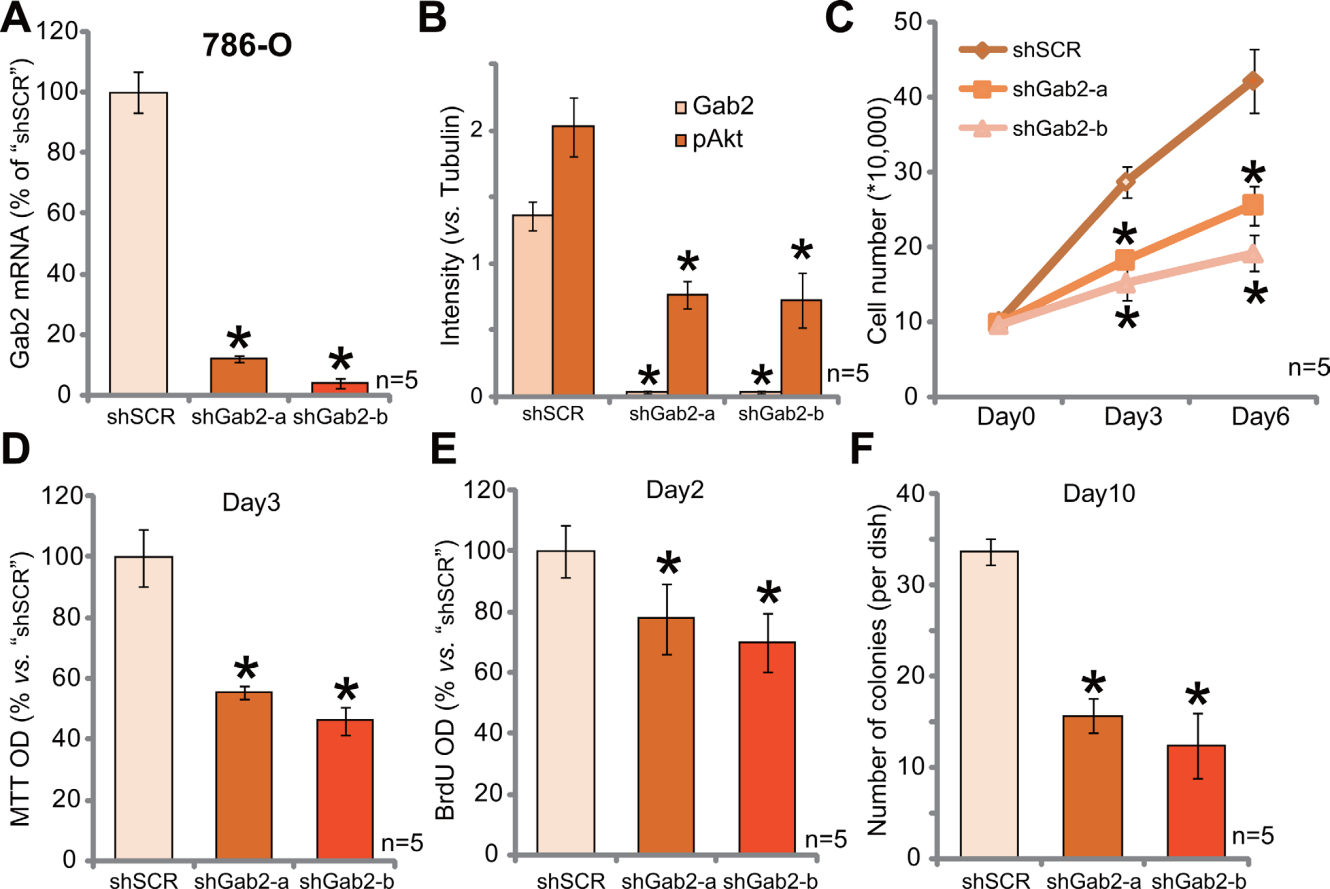
Gab2 shRNA knockdown inhibits Akt activation and 786-O RCC cell proliferation Expressions of Gab2 mRNA (**A**) and listed proteins (**B**) in 786-O RCC cells with indicated Gab2 shRNA (“shGab2-a” or “shGab2-b”) or scramble shRNA (“shSCR”) were shown; Cells were also subjected to the listed proliferation assays (**C**–**F**). At the beginning of these assays, exact same amount of viable cells with different background were plated (C–F). Gab2 and p-Akt expressions were quantified (*vs*. Tubulin, B). *p < 0.05 *vs*. “shSCR” group. Experiments in this figure were repeated five times, and similar results were obtained.

In order to study the function of Gab2 on RCC cell proliferation, shRNA method was applied. Two distinct lentiviral Gab2 shRNAs (with non-overlapping sequences) were applied, these two shRNAs were named as “shGab2-a” (from Santa Cruz) and “shGab2-b” (from Genepharm), respectively. qRT-PCR results in Figure 2A demonstrated that the applied Gab2 shRNAs indeed potently downregulated Gab2 mRNA in 786-O RCC cells. Consequently, Gab2 protein expression was also depleted (See quantified results in Figure 2B), along with significant Akt inhibition (See quantified results in Figure 2B). Remarkably, Gab2 shRNA knockdown significantly inhibited 786-O cell proliferation (Figure 2C–2F). Notably, cell proliferation was tested by following assays, including viable cell counting assay (Figure 2C), MTT assay (Figure 2D), BrdU ELISA assay (Figure 2E) and clonogenicity assay (Figure 2F). The two applied Gab2 shRNAs dramatically inhibited the number of proliferative cells (Figure 2C) the MTT OD (Figure 2D), BrdU ELISA OD (Figure 2E) and number of colonies remaining (Figure 2F). The application of scramble control shRNA (“shSCR”) had no such effects (Data not shown). These results imply that Gab2 shRNA knockdown inhibits 786-O cell proliferation.

### siRNA knockdown of Gab2 inhibits primary RCC cell proliferation

**Figure 3:**
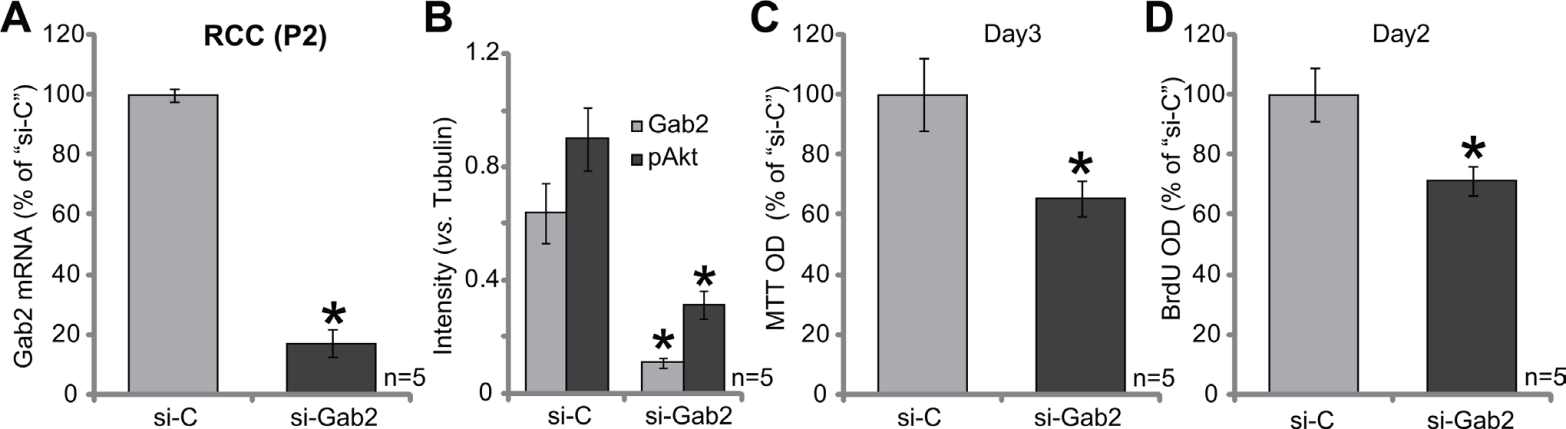
siRNA knockdown of Gab2 inhibits primary RCC cell proliferation Expressions of Gab2 mRNA (**A**) and listed proteins (**B**) in the primary RCC cells (P2), transfected with Gab2 siRNA (“si-Gab2”) or control non-sense siRNA (“si-C”), were shown. Cells were also subjected to MTT assay (**C**) and BrdU ELISA assay (**D**) to test cell proliferation. For the proliferation assays, exact same amount of viable cells with “si-C” or “si-Gab2” were initially plated (C–D). Gab2 and p-Akt expressions were quantified (*vs*. Tubulin, B). *p < 0.05 *vs*. “si-C” group. Experiments in this figure were repeated five times, and similar results were obtained.

We next tested the potential function of Gab2 in primary RCC cells. Gab2-targeted siRNA (“siGab2”, see Methods) was applied to knockdown Gab2 in the primary RCC cells [“RCC (P2)” line, see above]. As displayed, the application of the targeted-siRNA indeed silenced Gab2 in the primary cells (Figure 3A and 3B). As both Gab2 mRNA (Figure 3A) and protein (results were quantified in Figure 3B) were downregulated after siRNA transfection. Akt activation, reflected again by p-Akt (at Thr-308), was also decreased in Gab1-knockdown cells (results were quantified in Figure 3B). Importantly, MTT assay results in Figure 3C and BrdU ELISA assay results in Figure 3D demonstrated that Gab2 siRNA suppressed proliferation of the primary RCC cells. Thus, in the primary human RCC cells, siRNA-mediated knockdown Gab2 similarly inhibits Akt activation and cell proliferation.

### Gab2 over-expression facilitates 786-O cell proliferation

**Figure 4:**
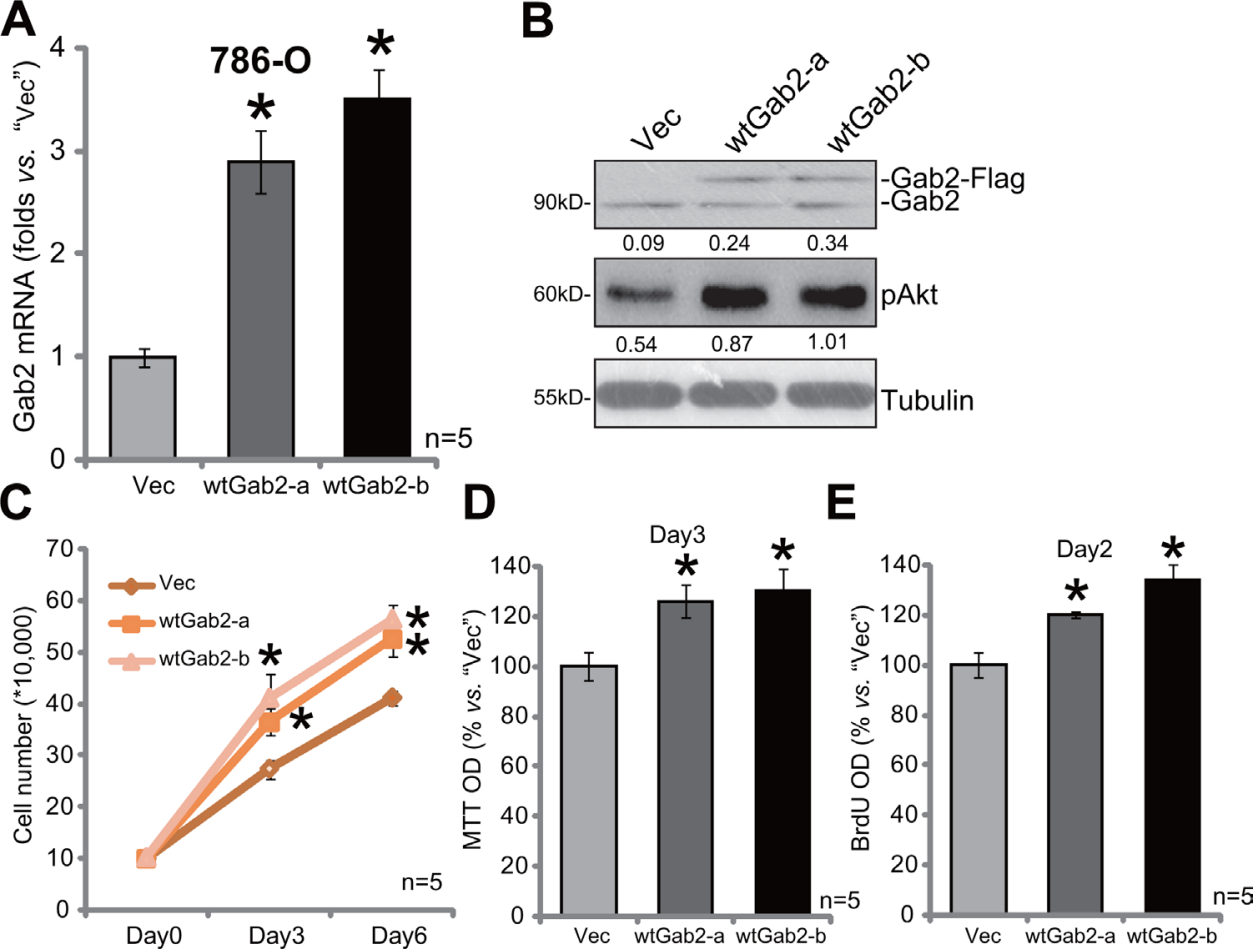
Gab2 over-expression facilitates 786-O cell proliferation Expressions of Gab2 mRNA (**A**) and indicated proteins (**B**) in the stable 786-O RCC cells, with wt-Gab1 (two lines, “-a”/“-b”) or empty vector (“Vec”, pSuper-puro-GFP-Flag), were shown. Above cells were also subjected to the listed proliferation assays (**C**–**E**). For the proliferation assays, exact same amount of viable cells with different background were plated (C-E). Gab2 and p-Akt expressions were quantified (*vs*. Tubulin, B). *p < 0.05 *vs*. “Vec” group. Experiments in this figure were repeated three times, and similar results were obtained.

To further support a function of Gab2 in RCC cell proliferation, we exogenously over-expressed Gab2 in 786-O cells. As described, the Gab2 (“wt-Gab2”, Flag-tagged) expression vector was introduced to 786-O cells. Via puromycin selection, two lines of stable 786-O cells with exogenous Gab2 were established, which were named as “wtGab2-a” and “wtGab2-b”. qRT-PCR assay results in Figure 4A confirmed Gab2 mRNA upregulation in the stable cells [as compared to the vector control (“Vec”) cells]. Gab2 protein expression and p-Akt were accordingly increased in the stable cells (Figure 4B). Remarkably, growth of these Gab2-over-expressing 786-O RCC cells was obviously faster than the vector control cells (Figure 4C). Meanwhile, cell proliferation, tested by MTT assay (Figure 4D) and BrdU incorporation assay (Figure 4E), was also facilitated with exogenous Gab2 over-expression. These results together demonstrate that exogenous Gab2 over-expression facilitates Akt activation and 786-O cell proliferation.

### Exogenous expression of miR-302c silences Gab2 and inhibits 786-O cell proliferation

**Figure 5:**
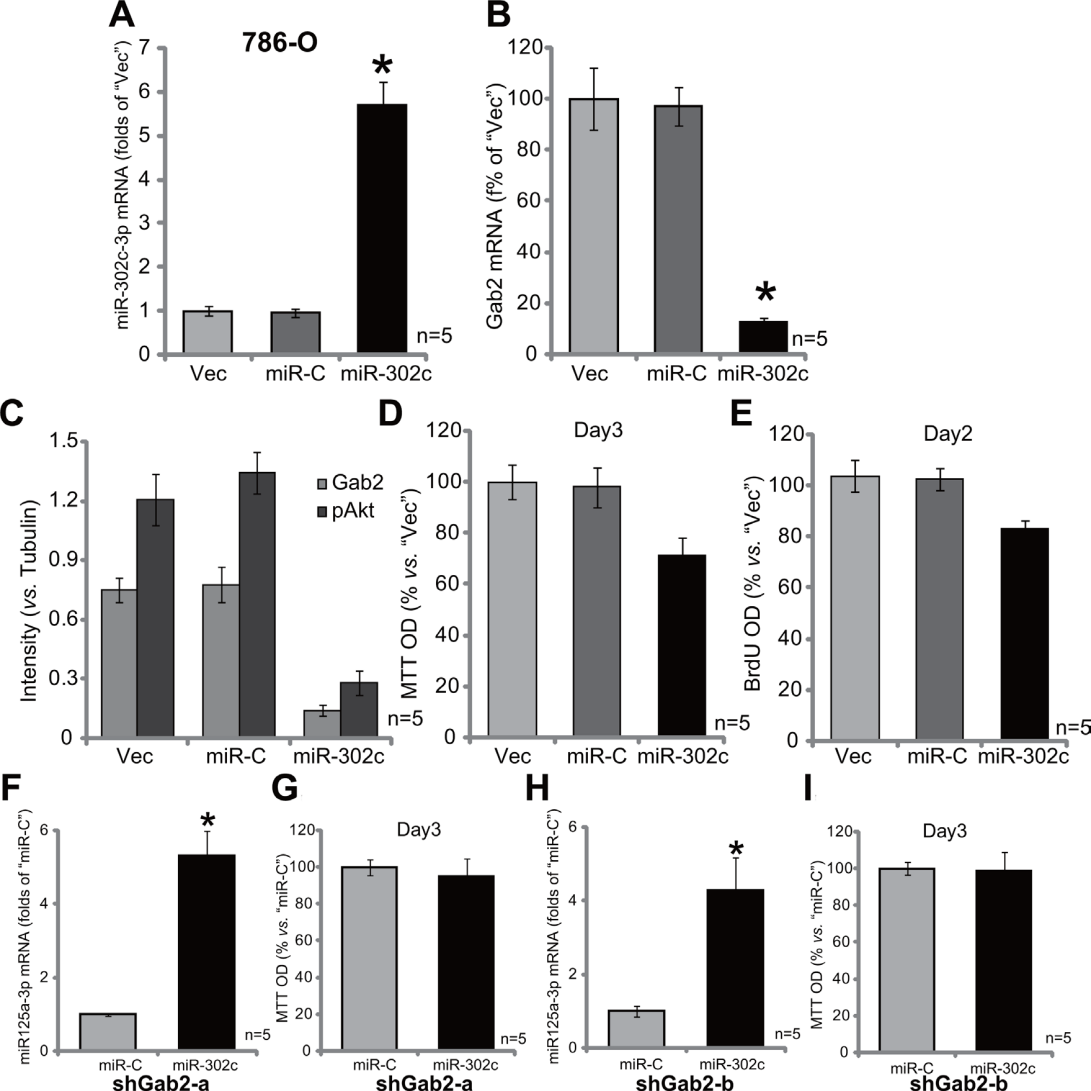
Exogenous expression of miR-302c silences Gab2 and inhibits 786-O cell proliferation Stable 786-O cells, expressing miR-302c, miR-control (“miR-C”) or the empty vector (“Vec”, pSuper-puro-GFP-Flag), were subjected to qRT-PCR assay of miR-302c-3p (**A**) and Gab2 mRNA (**B**); Expression of listed proteins was tested, and results were quantified (**C**); MTT assay (**D**) and BrdU ELISA (**E**) were also applied to test cell proliferation. Stable 786-O cells, with indicated Gab2 shRNA (“shGab2-a” or “shGab2-b”), were transfected with miR-302c or miR-control (“miR-C”), miR-302c-3p expression (**F** and **G**) and cell proliferation (MTT assay, **H** and **I**) were tested. For the proliferation assays, exact same amount of viable cells with different background were initially plated (D, E, G and I). Gab2 and p-Akt expressions were quantified (*vs*. Tubulin, C). *p < 0.05 *vs*. “Vec”/“miR-C” group. Experiments in this figure were repeated three times, and similar results were obtained.

miR-302c-3p is a presumable Gab2-targeting miRNA (See results in Figure 1A). Our results above demonstrated that Gab2 upregulation was correlated with miR-302c-3p downregulation in RCC tissues and cells (Figure 1A). We therefore wanted to know if miR-302c-3p expression could cause Gab2 downregulation in RCC cells. A miR-302cexpression vector was constructed and introduced to 786-O cells. Via puromycin selection, the stable 786-O cell line with miR-302c-expressing vector was established (See method). qRT-PCR assay results in Figure 5A demonstrated that the expression level of miR-302c-3p was indeed increased significantly in the stable cells. Significantly, expression of miR-302c caused Gab2 mRNA depletion in 786-O cells (Figure 5B). Meanwhile, Gab2 protein expression and Akt activation (p-Akt at Thr-308) were both decreased sharply with miR-302c expression (See quantified results in Figure 5C). Significantly, 786-O cell proliferation, tested by MTT assay (Figure 5D) and BrdU ELISA assay (Figure 5E), was also suppressed. These results indicate that miR-302c expression inhibits Gab2 expression, Akt activation and RCC cell proliferation.

If Gab2 is the primary target of miR-302c-3p, miR-302c-3p shall be in-effective in Gab2-silenced cells. We thus exogenously expressed miR-302c in the two lines of Gab2-depleted 786-O cells (See Figure 2). Remarkably, forced miR-302c expression (see miR-302c-3p over-expression in Figure 5F and 5G) failed to affect proliferation (MTT assay) of 786-O cells with depleted-Gab2 (by shRNA, Figure 5H and 5I). Thus, miR-302c-3p is very much invalid again Gab2-depleted 786-O cells, confirming that Gab2 could be the primary target of miR-302c-3p in 786-O cells.

### AntagomiR-302c depletes miR-302c, causing Gab2 upregulation, Akt activation and 786-O cell proliferation

**Figure 6:**
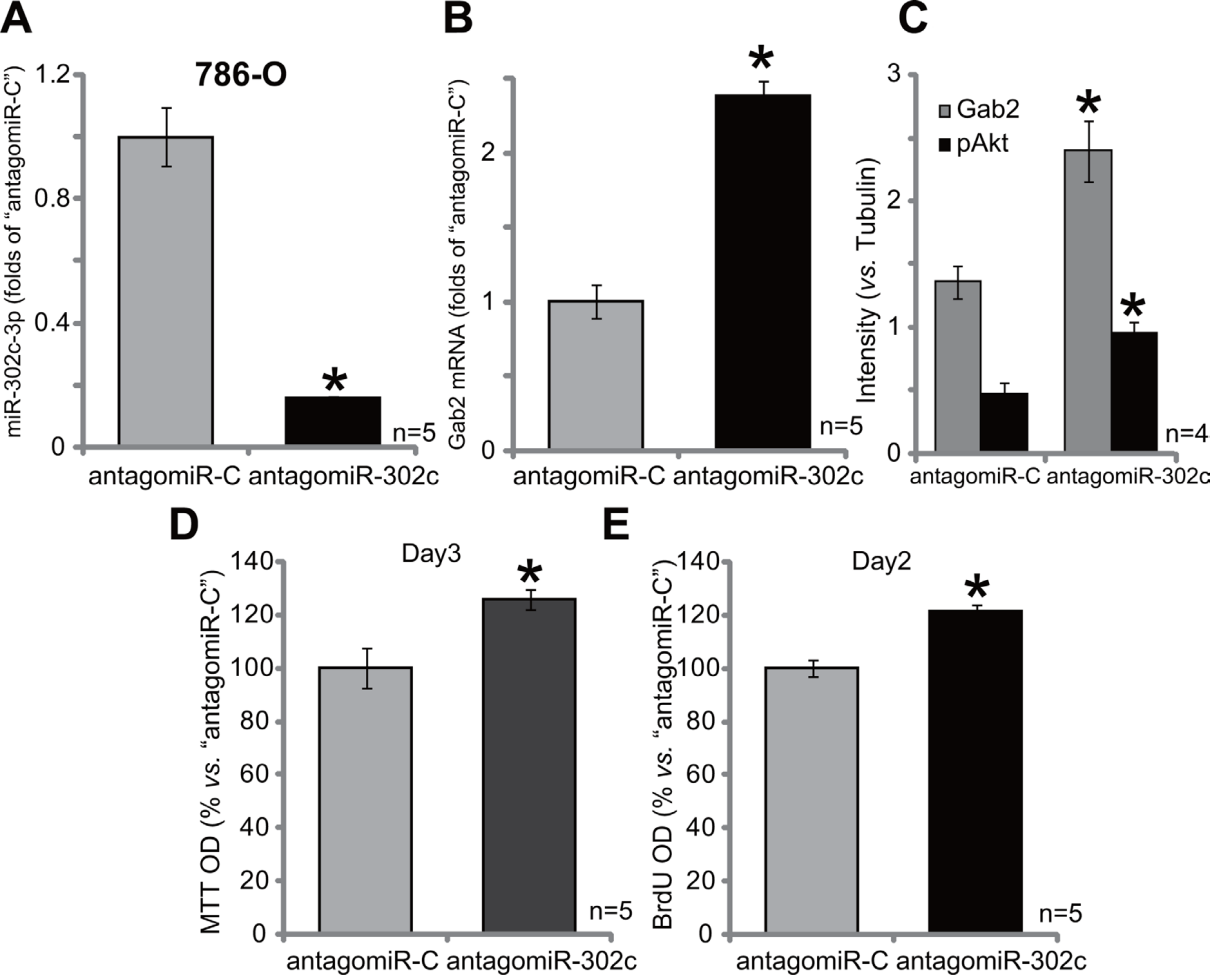
AntagomiR-302c depletes miR-302c, causing Gab2 upregulation, Akt activation and 786-O cell proliferation Stable 786-O cells, expressing antagomiR-302c or antagomiR-control (“antagomiR-C”), were subjected to qRT-PCR assay of miR-302c-3p (**A**) and Gab2 mRNA (**B**); Expression of listed proteins was shown (**C**); MTT assay (**D**) and BrdU ELISA (**E**) were employed to test cell proliferation. For the proliferation assays, exact same amount of viable cells with different background were initially plated (D–E). Gab2 and p-Akt expressions were quantified (*vs*. Tubulin, C). *p < 0.05 *vs*. “antagomiR-C” group. Experiments in this figure were repeated four times, and similar results were obtained.

At last, antagomiR-302c, the anti-sense miR-302c [27], was introduced to 786-O cells (See method). Expression of antagomiR-302c indeed led to significant miR-302c-3p downregulation in 786-O cells (Figure 6A). As a result, Gab2 mRNA expression (Figure 6B) was also increased. Gab2 protein expression and p-Akt were also enhanced in antagomiR-302c-expressin 786-O cells (See quantified results in Figure 6C). Consequently, 786-O cell proliferation, tested again by MTT assay (Figure 6D) and BrdU ELISA assay (Figure 6E), was increased by antagomiR-302c. The control non-sense antagomiR, or antagomiR-C, failed to affect Gab2 expression, Akt activation and RCC cell proliferation. Thus, antagomiR-302c depletes miR-302c, causing Gab2 upregulation, Akt activation and 786-O cell proliferation.

## DISCUSSION

Gab2 participates in signal transduction downstream of cytokine receptors, receptor tyrosine kinases, and antigen receptors [28]. It has been previously reported that aberrant Gab2 signaling is associated with human tumorigenesis [29–31]. Gab2 is involved in many cancerous behaviors, including cellular growth, survival, proliferation, and migration [28–31].

Our results imply that Gab2 could be an important oncogenic protein for human RCC. Gab2 expression, both mRNA and protein, was significantly elevated in human RCC tissues, which was correlated with Akt over-activation. Meanwhile, Gab2 upregulation and Akt activation were also observed in established and primary human RCC cells. Yet, their levels were low in the HK-2 non-cancerous cells. Knockdown of Gab2, by targeted shRNA/siRNA, inhibited Akt activation and RCC cell proliferation. On the other hand, forced over-expression of Gab2 led to Akt hyper-activation and increased RCC cell proliferation. Thus, Gab2 represents a potential novel oncogenic protein of RCC.

miR-302c could be involved in the regulation of multiple physiological and pathological processes. For example, Rosa *et al*., showed that miR-302 family members are involved in the differentiation of human embryonic stem cells [32]; miR-302c could directly target the estrogen receptor in human breast cancer [33]. Dysregulation of miR-302 is seen in biliary tract cancer and thyroid cancer [34]. Recently, Zhu *et al*., showed that miR-302c could inhibit hepatocellular carcinoma cell growth by targeting the endothelial-mesenchymal transition of endothelial cells [27].

We here proposed that miR-302c-3p depletion could be responsible for Gab2 upregulation and it-mediated RCC cell proliferation. miR-302c-3p level was decreased in both RCC tissues (*vs*. normal RCC tissues) and RCC cells (*vs*. HK-2 cells). Exogenous expression of miR-302c-3p caused Gab2 silence, Akt inhibition and proliferation inhibition in RCC cells. On the other hand, miR-302c-3p depletion by introduction of its anti-sense (antagomiR-302c) induced Gab2 upregulation, Akt over-activation and 786-O cell proliferation. Remarkably, miR-302c-3p over-expression failed to affect proliferation of 786-O cells with depleted-Gab2. These results imply that Gab2 shall be the primary target of miR-302c-3p in mediating its anti-RCC cell activity. More mechanistic insights studies were needed to support this notion.

## CONCLUSIONS

In conclusion, we provided evidences to imply that miR-302c-3p downregulation in human RCC cells causes Gab2 over-expression, Akt hyper-activation and cell proliferation. Gab2 could be a novel oncotarget protein of human RCC.

## MATERIALS AND METHODS

### Antibodies

All the antibodies utilized in this study were purchased from Cell Signaling Technologies (Beverly, MA).

### Human RCC tissues

As described [12], tissue specimens were from eleven (11) distinct nephroureterectomy RCC patients. All patients were administrated at the Second Affiliated Hospital of Nantong University (Nantong, China). The enrolled patients received no irradiation or chemotherapy prior to surgery. Tumor tissues and the surrounding normal renal tissues were separated and paired. Tissues were thoroughly washed, and then minced, which were then maintained in DMEM plus 10% FBS. Tissues were lysed and analyzed by Western blot assay and PCR assay. The protocols using human tissues were approved by the Ethics Review Board (ERB) and Internal Review Board (IRB) of Nantong University (Nantong, China). The written-informed consent was obtained from each enrolled patient. All investigations were conducted according to the principles expressed in the Declaration of Helsinki.

### Culture of established human cell lines

The culture of established human RCC cell lines (786-O and A489) as well as the HK-2 tubule epithelial cells was described previously [10–12, 35].

### Primary culture of human RCC cells

As described [12], RCC tissues were obtained from two primary RCC patients (See above. Patient 1, male, 54-years old; Patient 2, male, 43-years old). RCC tumor tissues were minced, and digested via collagenase I. Individual cells were pelleted, rinsed and filtered. The primary RCC cells were then cultured in the previously-described medium [12]. Two primary RCC cells lines, named RCC (P1) and RCC (P2), were established in this study. The primary RCC cells of passage 3–8 were utilized for further experiments.

### Methylthiazol tetrazolium (MTT) assay

Cell proliferation was tested by routine MTT assay. The detailed protocol was described early [10–12, 35].

### Clonogenicity assay

The protocol was described in our previous studies [10–12, 35]. Briefly, 786-O RCC cells with applied treatment/s were cultured for 10 consecutive days. Afterwards, the number of viable colonies were counted.

### BrdU ELISA assay

Cells with applied treatment were incubated with BrdU (10 μM, Cell Signaling Tech, Shanghai, China). BrdU incorporation was determined in ELISA format using the attached protocol. BrdU OD value of treatment group was normalized to that of control group.

### Western blot assay

As described [10–12, 35], cell and tissue lysate samples (40 μg per treatment) were fractionated on SDS-page gels, and were transferred to PVDF blots. The blot was probed with designated primary antibody, followed by incubation of the corresponding second antibody (Pierce). ECL was applied to visualize the interested band.

### Quantitative real-time PCR (qRT-PCR assay)

Total RNA was extracted via the SV total RNA isolation kit (Promega), and reverse transcription was performed through the TOYOBO ReverTra Ace-a RT-PCR kit (TOYOBO, Japan) [12]. cDNA was mixed with SYBR Green PCR Master Mix. Quantitative real-time PCR (qRT-PCR) was analyzed through the ABI-7700 system (Applied Biosystems). *Glyceraldehyde-3-phosphate dehydrogenase (GAPDH)* primers, forward, 5′-GAAGG TGAAGGTCGGAGTC-3′; reverse, 5′-GAAGATGGTGA TGGGATTTC-3′ [12]. *Gab2* primers, forward, 5′-CGAA GAGAACTATGTCCCTATGC-3′; reverse, 5′-AGGGGCA GGACTGTTCGT-3′ [36]. After amplification, melt curve analysis was performed to calculate product melting temperature. For normalization, *GAPDH* was utilized as the reference gene, and ^ΔΔ^Ct method was applied. The detection of mature miR-302c-3p was through the TaqMan microRNA assay of has-miR-302c-3p (Applied Biosystems, Shanghai, China) (see method in [37]). Twenty ng of RNA was reverse-transcribed by the TaqMan MicroRNA Reverse Transcription kit (Applied Biosystems) and the looped primer provided by the specific TaqMan microRNA assay.

### Gab2 shRNA

The two commercial-available non-overlapping lentiviral Gab2 shRNAs were obtained from Santa Cruz Biotech (sc-40606-V, Shanghai, China; “shGab2-a”) and Genepharm (#5631, Shanghai, China; “shGab2-b”), respectively. For infection, 786-O RCC cells were cultured in six-well culture plate of 50–60% confluence in the presence of polybrene (Sigma, 2.0 μg/mL). The lentiviral-shRNA was added to the cells. Virus-containing medium was replaced with fresh medium after 24 hours. Stable clones were selected by puromycin (0.5 μg/mL) for 10 days. Afterwards, Gab2 expression in the resistant colonies was tested by Western blot assay or qRT-PCR assay.

### Gab2 siRNA

To transiently knockdown Gab2 in primary human RCC cells, siRNA strategy was used. siRNA sequences for human Gab2 were combination of 5-CCTGAATGTGT GCCTTAAA-3, and 5-GCCAACTCTGTTCACGTTT-3 [29]. Gab2 siRNAs were synthesized by Genechem (Shanghai, China). A negative control scramble siRNA was described early [12]. siRNA (200 nM each, 24 hours) transfection was performed via the described Lipofectamine 2000 (Invitrogen, Carlsbad, CA) method [12].

### Gab2 over-expression

The full-length human *Gab2* cDNA (provided by Genepharm, Shanghai, China) was sub-cloned into pSuper-puro-GFP-Flag vector to generate Gab2 expression construct. 786-O cells were seeded onto six-well plates at 50–60% confluence. After 24 hours, cells were transfected with the Gab2 construct via Lipofectamine 2000 transfection reagent (Invitrogen) for 24 hours. Puromycin (0.5 μg/mL, Sigma) was then added to select stable cells (10 days). Gab2 expression in the resistant colonies was tested by Western blot assay or qRT-PCR assay.

### Exogenous expression of miR-302c and antagomiR-302c

A short hairpin structure against the hsa-miR-302c gene (miR-302c) (F: 5′-TTAAGTGCTTCCATG TTTCAGTGGTTCAAGAGACCACTGAAACATGGAA GCACTTATTTTTTC-3′, R: 5′-TCGAGAAAAAATA AGTGCTTCCATGTTTCAGTGGTCTCTTGAACCACT GAAACATGGAAGCACTTAA-3′) [27] was synthesized, annealed, and cloned into the HpaI and XhoI sites of pSuper-puromycin vector (pSuper-puro-miR-302c). The vector was then co-transfected with the packaging plasmids pCMV-VSVG and pCMV-dR8.91 via Lipofectamine 2000 to construct the viral particles in 293T cells. The infection of 786-O cells with the viral particles was performed. The infected cells constitutively expressed miR-302c. For permanent inhibition of miR-302c, vectors bearing an anti-miR-302c sequence (GCATTAACATGGAATTCCC, named as antagomiR-302-c) [27] was packaged into the virus.

### Statistical analysis

Data were expressed as mean ± standard deviation (SD). Statistical analyses were performed by one-way analysis of variance (ANOVA) with the GraphPad software. Significance was set at *p* < 0.05.
